# A Blockchain-Based Trust Model for the Internet of Things Supply Chain Management

**DOI:** 10.3390/s21051759

**Published:** 2021-03-04

**Authors:** Mabrook S. Al-Rakhami, Majed Al-Mashari

**Affiliations:** 1 Research Chair of Pervasive and Mobile Computing, Department of Information Systems, College of Computer and Information Sciences, King Saud University, Riyadh 11543, Saudi Arabia; 2 Information Systems Department, College of Computer and Information Sciences, King Saud University, Riyadh 11543, Saudi Arabia; majed@ksu.edu.sa

**Keywords:** supply chain, trust, blockchain, security

## Abstract

Accurate data and strategic business processes are crucial to all parties in a supply chain system. However, the absence of mutual trust can create a barrier to implementation. Several studies have shown that supply chains face challenges arising from a lack of trust with respect to the sharing of data. How well each party trusts the data they receive can have a profound influence on management decisions. Blockchain technology has been widely used to process cryptocurrency transactions. Recently, it has also proved to be effective in creating trust in the Internet of things (IoT) domain. Blockchain technology can facilitate mutual trust between parties who would otherwise have been doubtful of each other’s data, allowing for more effective and secure sharing of data. However, if the blockchain is not IoT-optimized, companies can experience significant delays and the need for extensive computational capacity. Moreover, there are still some limitations regarding the consensus between the nodes in the traditional consensus approaches. Here, we propose an alternative approach to creating trust in supply chains with diverse IoT elements. Our streamlined trust model simplifies data sharing and reduces computational, storage, and latency requirements while increasing the security of the IoT-based supply chain management. We evaluate the suggested model using simulations and highlight its viability.

## 1. Introduction

Blockchain technology is widely regarded as a revolutionary, peer-to-peer, decentralized option for data organization [1]. It allows for the formation of decentralized monetary systems such as bitcoin, smart contracts, and other resources that can be managed online, such as smart property. The technology was initially developed by Nakamoto in 2008, primarily to facilitate cryptocurrency transactions [2]. More recent studies have focused on how a blockchain can be used to distribute ledger systems and other financial transactions [2]. Blockchain technology allows different entities to exchange data and transactions in a few minutes, without intervention or verification by third parties. This can be accomplished through a shared data framework that utilizes computer algorithms to create real-time self-updates [3]. Blockchain technology can also settle financial transactions without mediation from banks and other trusted institutions. Blockchain technology promises to revolutionize how other organizational domains, including supply chains, conduct business [4]. Moreover, blockchain technology enables distributed data-exchange security, which can have a massive impact on organizational governance. It can also transform how parties in supply chains structure their relationships, and how they eventually exchange products and data [5].

Integrating blockchain technology with recent innovations, such as the Internet of things (IoT), can improve the creation of permanent records that can be shared and acted upon throughout a product supply chain [6,7]. Such integration can improve how businesses trace and monitor products, enhancing their legitimacy and authenticity. The overall result would be a marked improvement in efficiencies and the global economy [8].

Several researchers have suggested that businesses should use IoT components in supply chain management, including radio-frequency identification (RFID) [9], wireless sensor networks (WSNs) [10], a geographic information system (GIS) [11], and a global positioning system (GPS) [12]. Barcodes, RFID and GPS tags, sensors, and chips can be used to trace every step in the movement of shipping containers, products, and packages. Through the IoT, goods can be traced reliably and securely in real time [13]. One interesting proposal of a blockchain technology-based framework related to an e-commerce cross-border supply chain was published recently in [14]. The authors of this framework created an innovative multi-chain structured model based on the blockchain technology implementation. Moreover, they also introduced a wallet mechanism integrated within the key methods of distribution in the network. The authors of [15] also conducted a survey focused on challenges, applications, and open study opportunities. Their research reviewed the progress in blockchain technology within various fields, including healthcare, energetic technologies, finance, and some others.

We propose combining IoT and blockchain technology to solve the challenges of trust between supply chain parties and preserve data integrity. A blockchain offers a pathway to developing IoT technology that can facilitate the sharing of information that every party can see and trust. Each source of data is always visible, which makes the shared data secure and guaranteed. If large volumes of information have to be circulated among various members in various systems, such integration would prove useful.

Blockchain technology certainly has many benefits and advantages. Nevertheless, it also has some weaker points that must be considered too [16]. First, this technology is new and not fully developed yet, especially when it comes to its traceability within the supply chain. Secondly, the nodes still lack the complete consensus, which results in some functional limitations. The existing approaches towards the matter of consensus include protocols such as delegated proof-of-stake (DPoS), proof of work (PoW), and proof of stake (PoS), but none of them are perfect. Some of them (e.g., PoW) require computational power-demanding hardware, and the mining process is also accompanied by high energy consumption [17]. PoS protocol can, on the other hand, lead to undesired monopolization and centralization, as it selects leaders based on the percentage of stakes in their ownerships [18]. Similarly, DPoS protocol weighs votes based on the stake ownership of each involved node. This means that wealthy nodes can easily increase their influence over the whole network and create a monopoly. The issue of a few electable block producers poses yet another downside, as this can also increase the risk of centralization within the network [19].

The proposed trust model aims to solve several trust issues in a decentralized community where the nodes do not need to spend too much energy and waste computational power for performing blockchain activities. Precisely, we propose using blockchain technology to create a lightweight trust model that will create the minimum requirements needed for participants in a supply chain to trust the data they receive.

The contribution of this work is threefold:

- We demonstrate how blockchain can integrate with IoT-based supply chains.
- We propose a lightweight trust model in which blockchain and IoT work together to enhance information sharing among supply chain parties.
- We conduct extensive simulation experiments that highlight the effectiveness and security of the proposed model.

The rest of this paper is structured as follows. Section 2 of this paper introduces some preliminary concepts. Section 3 presents the proposed trust model in detail. Trust model evaluation is conducted in Section 4. Performance analysis of our lightweight trust model is discussed in Section 5, which is followed by concluding observations in Section 6.

## 2. Preliminaries

### 2.1. Blockchain Technology

Distributed ledger technology or a blockchain can be defined in a variety of ways. The most common definition for blockchain is a decentralized database in which transactions are recorded using a virtually unmodifiable cryptographic signature [20].

**Figure 1:**
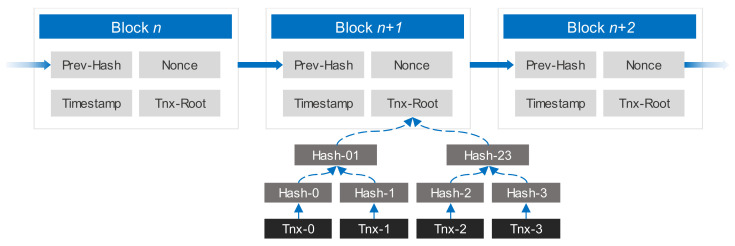
Blockchain structure.

Records can continuously be added to the decentralized database to create blocks, which are protected against manipulation and alteration [21]. Each block is connected to the previous block and has a timestamp. Figure 1 shows the structure of a standard blockchain. Different entities can create smart contracts based on a blockchain, without intervention from humans. The data contained in such a contract is difficult to alter, as blocks cannot be changed after they have been formed. We define smart contracts on a blockchain as any actions that are implemented on contributing nodes, creating mutual agreement in the results of such transactions without the need for third-party mediation of information and money.

After defining these fundamentals, we classified a blockchain as both permission-less and permissioned [22]. In addition, numerous other consensus mechanisms are available, including proof of authority and proof of work (PoW) [23].

**Figure 2:**
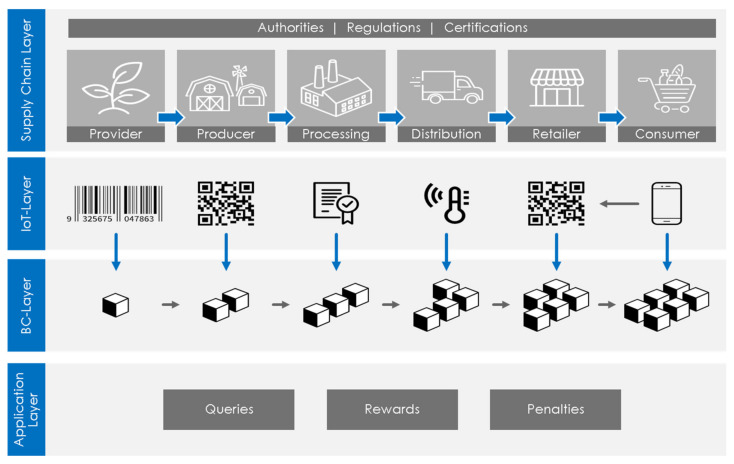
A blockchain Internet of things (IoT)-based supply chain system.

Figure 2 illustrates a typical blockchain structure for an IoT-based supply chain. Peers can only enter the system using a unique set of public-private key pairs. Blocks consist of a body and a header. The body comprises transactions that a user signs using a private key. A public key is used to verify the digital signature. The block header contains basic information about the block, including timestamps, version numbers, block size, and transaction numbers, among other elements. Merkle hash trees are often used to generate hash values for every transaction in a block to limit the chain’s storage overheads. Blocks also include hash values from previous blocks to connect a chain of blocks.

Once each block is created, it is distributed to miners, whose role is to validate every transaction. Once a transaction in the block is approved, a consensus protocol, for example, a PoW, is executed by locating a nonce that makes the block’s hash begin with a defined number of zeros. The block can then be added to the blockchain and be disseminated to every node in the structure. Blocks that are already in the chain accept the new block by integrating its hash into the recently created block.

### 2.2. Blockchains for Supply Chain Management

Blockchain integration with supply chain management may lead to revolutionary changes in different industries. Traditional methods of conducting businesses are being revisited, mainly to reduce the need for human mediation in transactions [24]. Using the principles of a food chain supply as an example, Figure 2 illustrates how smart contracts perform to clarify and interpret the relations between the blockchain technology and the Internet of things within the supply chain. Provider represents the first entity within the supply chain, whereas consumer is the last one. The network that connects these entities consists of the Internet of things.

Both parties negotiate and agree on conditions. Once both parties’ conditions for the exchange are fulfilled, a smart contract is created, coded, and archived in a blockchain structure. All data is recorded and managed through the use of IoT devices such as RFID, quick-response codes, and WSNs. Once the contract is created, goods and funds are transferred according to the terms of contract. No human mediation is required, and the transaction can proceed faster, at less cost. As all participants in the network have a copy of every transaction record, there is improved mutual trust.

Blockchain technology, therefore, offers numerous advantages that can potentially boost supply chain management in a variety of ways.

- Entities can see and audit transactions in a system through an entire life cycle of production, delivery, maintenance, deployment, and retirement. The blockchain also provides for the monitoring and chain of custody of all field devices throughout the life cycle.
- System hardware, firmware, and software component records are not archived on a single server vulnerable to deletion or alteration of data. Instead, a cryptographic metadata hash makes it possible to see the current and past patch information from mutually agreed blockchain data.
- Accessing and viewing supply chain data is easier, which enhances and accelerates cooperative systems between vendors.
- Intermediary brokers who can be vulnerable to manipulation are replaced by a blockchain that can enhance supply chain process security.
- Tracing principal parts through the entire system’s life cycle allows for effective engineering processes.
- The blockchain consensus algorithm will flag any unpatched field devices, blocking any malicious changes in the default field device configuration. This allows for enhanced monitoring of digital resources, device security, and compliance.

While blockchain technology shows great potential in supply chain management, its widespread use in the industry is still at an early stage [25]. Blockchain technology is still developing a common definition, and this creates a variety of policy-related challenges. Rapidly shifting and disputed vocabulary regarding blockchains has led to challenges for regulators who are tasked with understanding the technology. Poorly defined vocabulary can also prevent regulators from using blockchain technology and offering suggestions on how it can be improved.

A common source of confusion in blockchain-related definitions is the perception that the technology is the same as bitcoin. Although blockchains may include publicly recorded cryptocurrency transactions, permissioned or private blockchains usually do not involve monetary transactions. They also do not create public records.

In [26], a blockchain was described as a public digital ledger in which cryptocurrency transactions are recorded. In a similar manner, a blockchain has been defined as a decentralized cryptocurrency transaction ledger [26]. These definitions may prove contradictory in different industries. They may also differ as a result of the roles they are required to fulfill or the technology they use. Zero-proof, PoW, burn, or authority are some ways in which consensus algorithms that set up trust mechanisms for a distributed ledger data security can be described [27].

### 2.3. IoT and Supply Chain Management

Considerable research efforts have been devoted to how IoT technologies, such as RFID, GPS, WSN, and GIS, can be used in supply chain management [28]. Researchers have attempted to use such traceability equipment in a variety of ways. One example is the rule-based decision system proposed by Wang et al. [29] to monitor the distribution of agricultural products in real time. In [30], the author created a model that allowed entities to trace products in a supply chain. The model was based on the hypothesis that it has to collect, save, and distribute information through the entire supply chain using a blockchain and IoT systems. A study by Grunow and Piramuthu [31] created a model that used RFID technology to move perishable products from the supplier to the retailer and consumer’s perspectives. A key finding of their study was that RFID technology had the potential to benefit every party in a supply chain of perishable goods.

In [32], the authors proposed using RFID technology to trace and maintain animal food safety. Because the active distribution of data required the use of decentralized information servers, such servers used object names and discovery services in the framework. In [9], Tian developed a system to maintain a high level of product safety and quality in a Chinese agri-food supply chain, addressing several challenges in the existing centralized system using RFID and blockchains. A decentralized system was then developed to trace the movement of information in the supply chain.

Although many trust models have been proposed for supply chains based on blockchains and the IoT, a lightweight model is still required to guarantee secure and efficient management for supply chain transactions. Additionally, the proposed trust model aims to solve several trust issues in a decentralized approach where the nodes do not need to spend too much energy and waste computational power for performing blockchain transactions.

## 3. Proposed Trust Model

Supply chain management systems perform a crucial role in different activities of different industries. The main goal of supply chain management is to increase customer value and ensure sustainable competitive advantage. Supply chain management relies on each partner in the loop, starting from supplier and ending at the manufacturer and beyond. Accordingly, supply chain systems are expansive, complex, and require trust insurance. It is crucial also to transmit information smoothly between the partners in the supply chain system. In this regard, IoT technologies can help to improve the timely responses and information storage. On the other hand, to ensure the security and transparency of the supply chain transactions, blockchain technology is an appropriate choice, considering the efficiency and effectiveness requirements that are required for supply chain systems.

**Figure 3:**
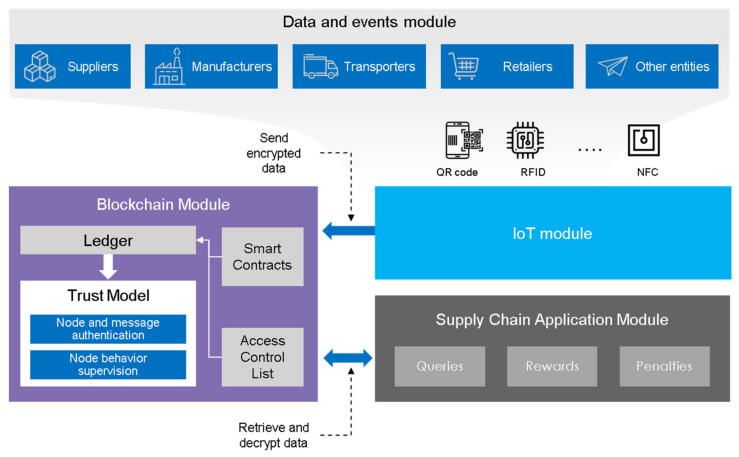
Conceptual diagram of a blockchain IoT-based supply chain with the proposed trust model.

In order to provide a trust model for the integrated IoT-based supply chain with blockchain, our proposed trust model is shown in Figure 3. The conceptual model in Figure 3 depicts a blockchain supply chain based on the IoT and the suggested trust model. The key data providing coherence and traceability of the events within the depicted supply chain (including the key product data, sensor data, and data from some additional sources) should be logged in a way that prevents any possible tampering with the data. This can be provided by the deployed blockchain technology. The model we propose verifies all the logged information and reproduces the true observation of the entities within the supply chain, utilized sensors, and other sources of data, which is essential for establishing trust in the original information and ensuring that the blockchain-logged data will be considered trustworthy.

Standardly, supply chains contain numerous types of products and entities. The trust should, therefore, consider all these various elements and their mutual interaction. The whole procedure can also be fully automated, providing real-time tracking and traceability within the process. For this purpose, our framework suggests the use of a trust model that integrates the blockchain technology, which can verify the reliability and accuracy of the data and provide a score-based evaluation for products and entities within the chain supply. We are also using smart contracts—independently operating software applications—to provide the automatization of the process. These programs are initiated when the process meets some preset requirements.

Our model comprises three key modules: Data, IoT Network, and Blockchain and Supply Chain system. The first of the modules contains data produced by sensors within the supply chain and trade events in between its nodes. The supply chain has an application layer with a database that can be used to store this raw data, while the cryptographically edited information (message digest) is passed on to the blockchain layer through the IoT module as a transaction. Such transactions are logged, stored, and processed at the blockchain according to the predefined rules for access; the access control list (ACL), which defines who has the right to write and read the data stored at the ledger.

In the next step, our proposed trust model authenticates and supervises the message and node. Subsequently, the blockchain and supply chain modules communicate with each other through a series of queries. Administrators can request the calculated value of trust scores on involved products and entities. According to the score they receive, they can publicly reward or penalize the involved parties by revoking them from the whole network.

The following section will focus on how our proposed trust model verifies and authenticates the message and nodes. We will also discuss further how the mechanism of rewards and penalizations works.

Entity in our framework stands for a party that conducted the transaction within the supply chain based on the IoT. *S* stands for the “source entity”, whereas *D* stands for the “destination entity”. The interplay between these two entities is used as an introductory model for the process of the transaction. Here is a simple summarization of the transaction process:

1. At first, node *S* performs an interaction request towards node *D*. The nodes subsequently query mutual trust score values (denoted as ℌ). With the increasing number of processed transactions, Internet of things devices within the blockchain will hold on to the trust score values ℌ, depending on the total number of failed and successful authentication attempts the respective nodes have carried out. The formula for the trust score value calculation goes as follows:
  (1) $$ℌ=\frac{\sum_{i=1}^{n}v}{n}$$
  The *v* stands for the evaluated value of the *D* node in the course of n counts of transactions. To proceed with the interaction, the trust score values for both of the nodes must exceed the given threshold.
2. Node S measures value ℌ according to Equation (1) towards D based on the records of their historical interaction (as depicted in Table 1).
3. The recommendation requests are subsequently sent by the *S* node to the third recommender node in order to get the trust value score on node *D*. The third recommender nodes are expected to make the decision based on the current situation and circumstances.
4. Based on the trust data provided by the third recommender node, we can calculate the indirect trust value as follows:
  (2) $$I=\frac{{ℌ}_{i}}{\sum_{j=1}^{n}{ℌ}_{j}}$$
5. Node *S* carries out the transaction with node *D*.
6. Subsequently, node *S* reviews its satisfaction with node *D*’s service, based on their mutual interaction. Node *S* automatically updates the trust value of the recommendation nodes based on this experience.

**Table 1:** Log record for related nodes’ trust values.

Node ID.	Interaction Number	Interaction Time	Trust Value
NodeID	IntNo	T	ℌ

### 3.1. Node and Message Authentication

Initializing IoT-based supply chain nodes requires the transmission of messages that are authenticated using asymmetric key cryptosystems. A random number is generated, and an RSA key is used to encrypt the number and create a time stamp. The time stamps will be required to decrypt random numbers before they are compared to query results from the hash function. Only if the random number from the hash query is identical to the one from the decrypted random number is the node verification considered successful.

### 3.2. Node Reward and Punishment

In our model, trust can be measured by evaluating the success/failure verification attempts in previous transactions and using the success/failure rate values in future transactions. The higher the success verification attempts of past encounters, the greater the trust. The trust value is then used to punish or reward nodes based on their performance. The trust value that each node has is a key indicator when trying to identify suspicious nodes within the IoT-based supply chain system, because if an individual node behaves abnormally, its specific trust value is affected negatively. The double threshold of each trust value (α,β) is used to determine the behavior of each node, according to the following Equation (3):

(3) $$Selected\;node\;\left\{\begin{array}{c} honest,\;if\;(ℌ<a)\;\\ suspicious,\;if\;\left(a\leq ℌ\leq \beta \right)\\ malicious,\;if\;(ℌ>\beta )\; \end{array}\right.$$

When the network is operating, each node is punished or rewarded with a higher or lower trust value, depending on how accurately it evaluates other nodes. If a node is highly accurate, it will be rewarded with a higher trust value. The opposite is true for highly inaccurate nodes. Therefore, whenever a node evaluates another node, its own trust value will change. In our trust mode, each node manages its own trust values; a simple historical interactive log record with other nodes on each interaction time is shown in Table 1.

Verification mechanisms are used to prevent inaccurate judgment of nodes. Nodes are therefore supervised when their trust values are lower than α. If they exhibit no abnormal behavior, their initial personal trust values are restored. If they exhibit unusual behavior, the trust value will be set to zero. As a way to ensure that malicious behavior is punished quickly, the total number of punished nodes is continually recorded. If the number increases, so does the severity of the punishment, with each iteration. For example, if the initial penalty for malicious behavior is 2, then $\left(x=x^{1}\right),\;\mathrm{thus}\;x=2$. The subsequent penalty would be $\left(x=x^{2}\right),\;\mathrm{thus}\;x=4$, and the third $\left(x=x^{3}\right),\;\mathrm{thus}\;x=8$. Penalties continue on this trajectory until the malicious node begins to be supervised.

### 3.3. Node Behavior Supervision

How node behavior is supervised and evaluated depends on the particular implementation network for each blockchain. Neighboring nodes (IoT devices) continuously oversee each other’s behavior on the blockchain. Each node’s behavior is therefore decided by real-time communication with its peers. Depending on the criteria for evaluation, the determination of each node’s trust level is affected by how it evaluates both itself and its peers. A source node is evaluated by the node with which it exchanges data or the transaction node.

If node *D* appraises source node *S*, we can denote the evaluation as $D_{i,j}$, which is also the ratio of the distance between nodes *S* and *D*. The transmission time can be calculated using the following formula:

(4) DS,D=aS,D∑p=1qtpk−tplq

The average time $t_{x}$ that it takes for a node to receive a response packet after it transmits a request packet is known as the bidirectional transmission time, while $a_{S,D}$ is the true distance between nodes *S* and *D*, q represents how many nodes are in the system, and $t_{pk}$ denotes the fastest travel time for the arbitrary probe packet. The value $t_{pl}$ is the fastest packet transmission time that relates to $t_{pk}$.

## 4. Trust Model Evaluation

In this section, we elaborate how the trust model is evaluated. Specifically, we used an attack model to test the efficiency of the proposed trust model. This section also describes the experimental settings and the practical analysis of the model in terms of behavior when faced with different attacks. We conclude with a practical evaluation of the scenarios covered.

### 4.1. Attack Model

We evaluated the trust model under different attack scenarios. Some of the scenarios include those executed by malicious nodes in a network. These are known as Sybil nodes, which will often undertake fake transactions in a distributed system.

- Attack Mode 1: In this type, one node in a network will try to subvert the system by sending fake transactions to local memory pools of other nodes. The fake transactions portion of the memory pool is inculcated by creating blocks that are distributed to others for the sake of validation before it can be added to the main blockchain.
- Attack Mode 2: In this mode, the creator node attempts to subvert the system by introducing fake transactions to a random block then relaying it to other nodes in order to validate the transaction. The rest of the nodes will perform the validation process and add the fake transaction to the main blockchain network.

### 4.2. Experiment Settings

We created a prototype model to demonstrate the functionality of the proposed model using virtual machines that are created on a personal computer. We evaluated the model on the basis of other traditional consensus defense protocols, such as proof of work (PoW), proof of stake (PoS), and delegated proof-of-stake (DPoS).

A number of assumptions were made in simulating the attack in the experimental setup:

- Each of the contributing nodes comprised 1 k transactions, which were stored in the nodes’ memory pool.
- The ratio of rogue nodes keeps changing in each scenario.
- The amount of fake transactions in the 1000 transactions is scaled up in each scenario, from 100 to 900.
- Every block has one transaction.

### 4.3. Node Trust Evaluation

Trust evaluation is conducted when the client node initiates a transaction and requests endorsements from neighboring nodes. The client node must send transaction proposals to available endorsing peers before a transaction can be fully entered into the ledger. The endorsing peer nodes will then evaluate the initiating node’s trust score based on a pre-existing endorsement policy, using a chain code. Different system thresholds are defined by the corresponding endorsement policies. The peer nodes evaluate how trustworthy the initiating node is by performing device classification. The trust scores and ratings of each device are then calculated and updated. Each device’s historical behavior is also considered when classifying the maximum probability of its trustworthiness.

### 4.4. Results

The simulated attacks were run against the trust model, PoS, PoW, and DPoS, to determine the ratio of correct and fake blocks for the consensus protocols under investigation. We increased the number of fake transactions and the ratio of malicious nodes in the course of the experiment to study the performance of these protocols.

**Figure 4:**
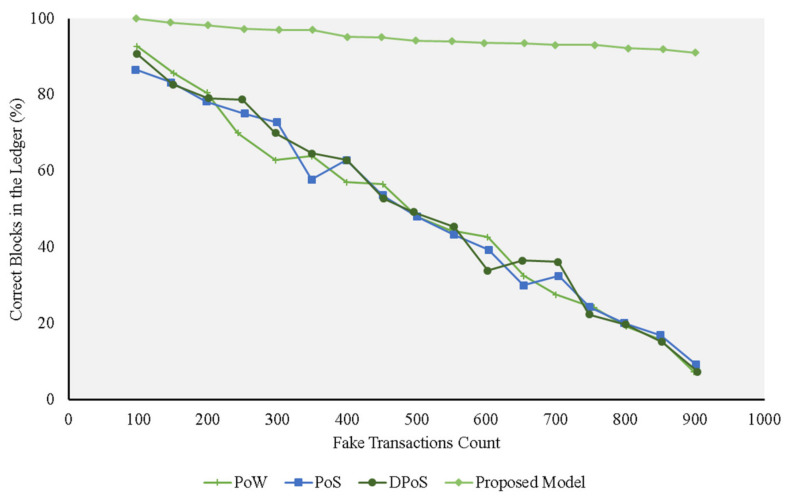
Experiments for different protocols on the fake transaction dataset (0% malicious nodes) compared with the proposed model.

Figure 4 depicts a scenario in which all the nodes are secured. In this case, we assumed that there were a various number of transactions in each node. The results showed that the trust model added the accurate transactions in the blockchain ledger, regardless of the number of rogue transactions in the nodes’ local memory pool. This can be explained by the fact that the model assigned trust scores for every transaction on the basis of where it originated. It only added transactions that met a certain trust score threshold (at least 80%). This way, the model filtered any rogue transactions and only added legitimate ones to the blockchain ledger. A comparison of our proposed trust model performance and performances of the alternative models is shown in Figure 4. As this figure illustrates, each of the alternative protocols struggled to differentiate genuine transactions from the fake ones. The DPoS consensus algorithm leverages similarity of the transactions, which means that if it encounters an increased number of false transactions, they are more likely to pass the checks. As a result, the ledger is gradually filled with fake blocks.

**Figure 5:**
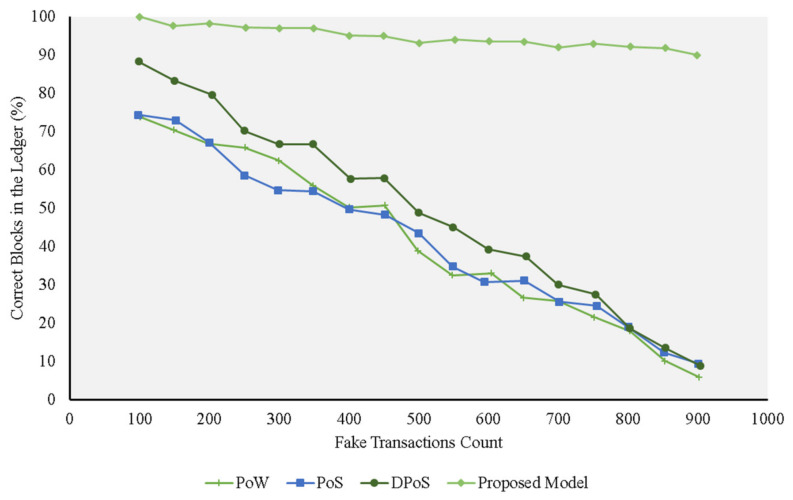
Experiments for different protocols on the fake transaction dataset (20% malicious nodes) compared with the proposed model.

**Figure 6:**
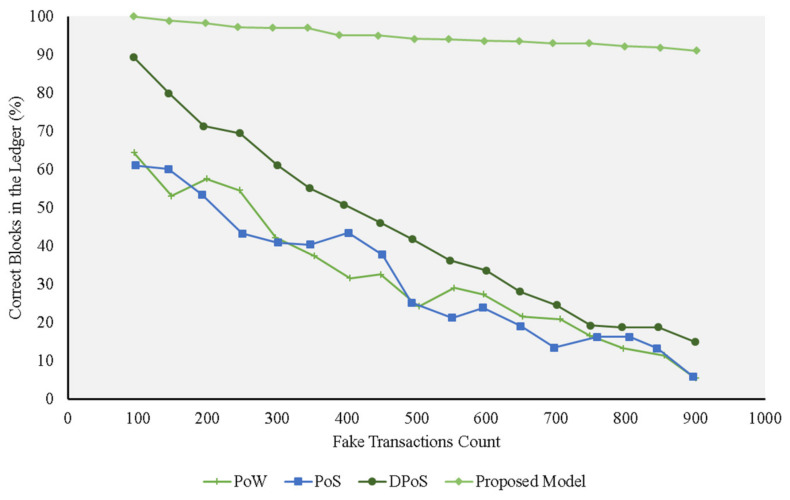
Experiments for different protocols on the fake transaction dataset (40% malicious nodes) compared with the proposed model.

**Figure 7:**
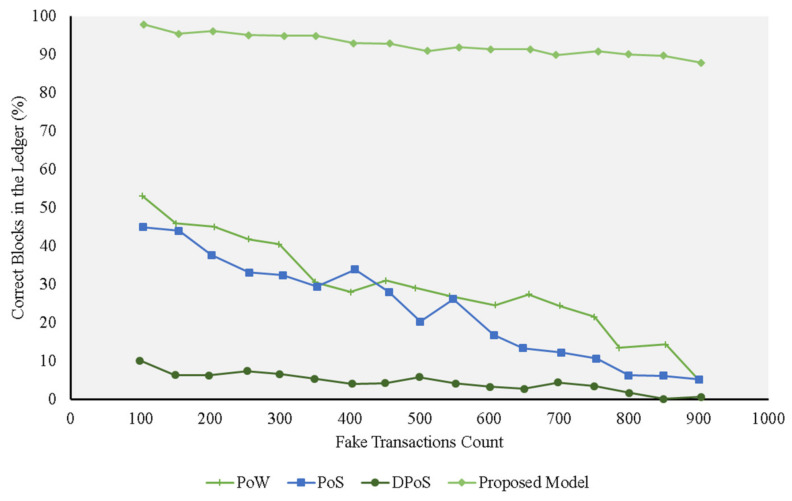
Experiments for different protocols on the fake transaction dataset (60% malicious nodes) compared with the proposed model.

Figure 4, Figure 5, Figure 6 and Figure 7 represent the presence of illegal nodes, which can take control of the way blocks are created and validated. Figures show that in terms of performance, the trust model outperformed the other consensus protocols. Our model became more resilient as the number of malicious nodes increased because the existing protocols relied more on computing power and simple selection approaches and did not consider trust in the block creator node. If a rogue node was designated as a block creator, then it creates the block, passes the cryptographic challenge, and relays the block to other nodes. The other nodes simply validated the hash values and the corresponding keys of the block and accepted it without factoring in the trustworthiness of the block, the creator, and the transactions undertaken. When the number of malicious nodes increased, the chances of picking a malicious node also increased, and the performance of all the traditional consensus protocols declined. In the case of the PoS protocol, for example, its performance was on par with the others, but it deteriorated when more malicious nodes were added to the network. This can be explained by the fact that it used similarity checks. Whenever the number of nodes increased, the chances that the rogue nodes had the same fake transactions for a given similarity check also increased. The protocol resulted in a high number of fake transactions in the blockchain.

The DPoS protocol, on the other hand, depicted good performance compared with the other existing approaches at the start (Figure 5 and Figure 6). This can be explained by the fact the algorithm used a voting approach to pick a block creation leader. This worked as long as the number of malicious nodes was lower than or equal to 50% of the nodes in the network. The probability that a good node was selected as the block creation leader was high, resulting in a high number of correct blocks in the network.

## 5. Performance Analysis

We simulated our proposed trust model to prove that the concept is viable, and to test and assess its effectiveness. This section provides more insight on the results of our analysis. We focused on the model’s computing power, latency, and memory requirements.

### 5.1. Evaluating the Proof of Work

The proof of work (PoW) concept describes an algorithm that keeps the blockchain network secure by requiring a requester to do some work before receiving feedback. Bitcoin is the most common example of a blockchain network that runs using PoW. Each node in the network computes a hash value, also known as a nonce, in the header to create consensus among users. Once blocks are distributed to miners, they will try to approximate a secret value and insert it into the block. The data contained in the header of the block is then combined and waits for encryption using a SHA-256 hash function. The new block is added to the blockchain by the miner that first acquired a hash function with an output beneath a certain threshold. The hash values become more difficult the more they are created to limit their generation. The hash functions begin with a number of zeros, which are currently at a difficulty level of 18. Because the hash value for each block cannot be chosen or predetermined, miners have to crack the puzzle by trying several combinations. To show the PoW consensus’ disadvantages, we implemented it using JavaScript to recreate the increase in difficulty. We used a computer with a Core i7-7820HK, 2.90 GHz processor, and 16 GB of RAM to simulate the appending of one block.

**Figure 8:**
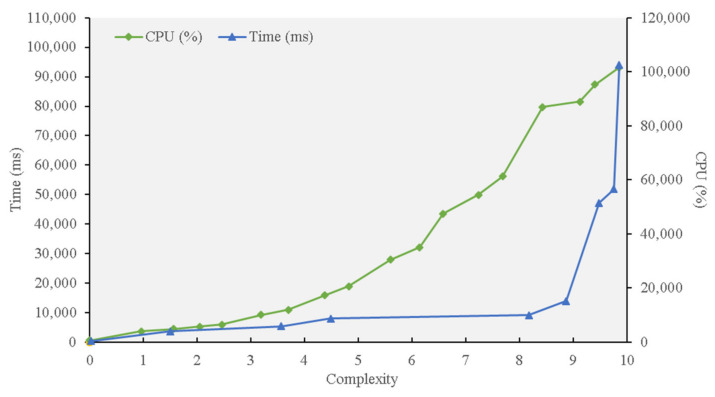
PoW Processing time and complexity experiment.

Figure 8 illustrates the results for the required computational power and time of PoW. The simulation results demonstrate that it is less efficient to apply the PoW consensus model in supply chain management.

### 5.2. Performance Evaluation

In this section of the study, we evaluate how our model performs compared to the PoW consensus. We fixed the level of difficulty at four to evaluate the required computational power, processing time, and memory requirements. We simulated adding 10 blocks to the blockchain three times and recorded the central processor evolution time.

**Figure 9:**
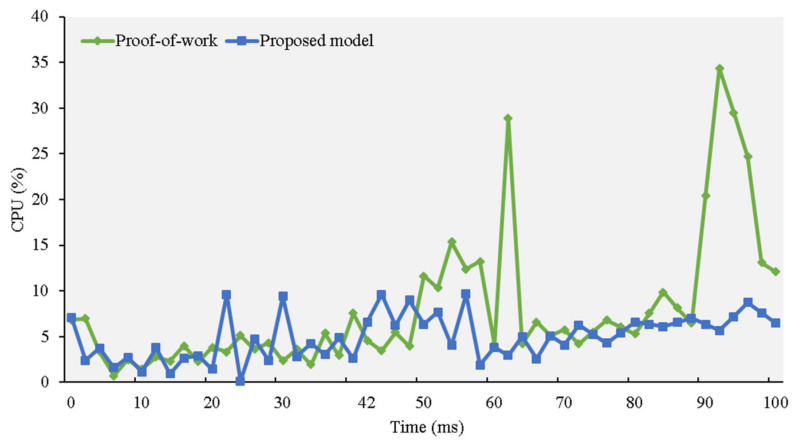
Computational power experiment.

Figure 9 illustrates the computational power required by the two consensuses. To measure the effectiveness of our proposed model, we added and removed blocks during the simulation. The algorithms required for blockchain mining can require substantial amounts of memory, and the system cannot provide the amount of RAM that consensus algorithms usually require. We found that a well-equipped computer is required to mine and crack the complex PoW puzzles by comparing how both consensuses used memory.

**Figure 10:**
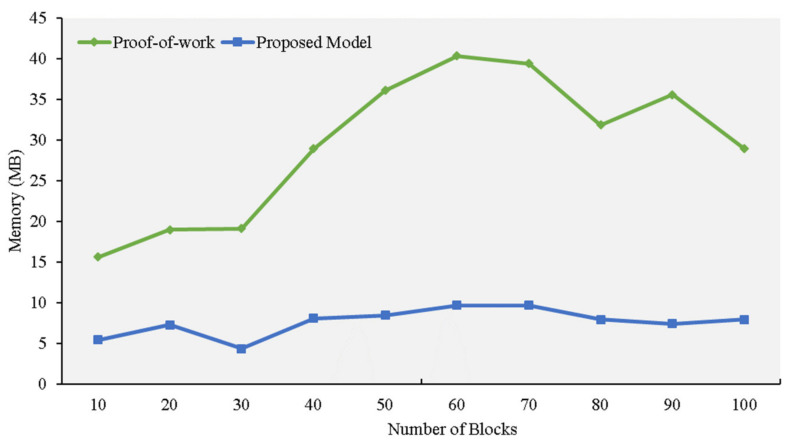
Memory evaluation experiment.

Even if the algorithm makes it appear that computational power is a more important factor than memory, large RAM requirements will slow down the mining process and increase delays. At 0.41% per block, our proposed model has a lower rate of memory growth, which will improve the block verification process (see Figure 10).

**Figure 11:**
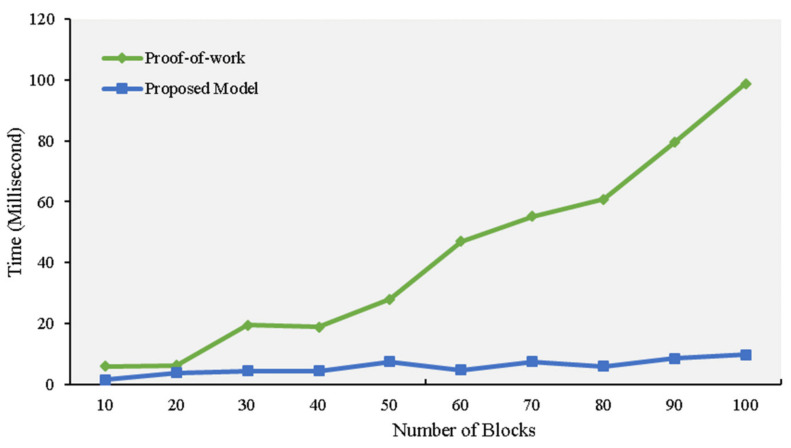
Delay evaluation experiment.

Figure 11 depicts the delay time for when a consensus algorithm adds blocks to the ledger. The PoW’s delay evaluation is shown with our model. As the figure illustrates, the algorithm for PoW slows down as more blocks are added to the chain. The algorithm required 4.2 s to add 10 blocks, while our model needed 0.58 s to complete the same process.

## 6. Conclusions

A blockchain-based supply chain system that is combined with IoT devices does not require trusted intermediaries but establishes trust between transacting entities using a different approach. Such a system can be used to track, trace, and manage products throughout a supply chain. Data can therefore be shared securely between entities that otherwise would have doubted the accuracy of each other’s data. IoT devices typically tend to be compact, which poses a challenge, given that blockchain processing inevitably requires considerable computational power. The trust model that is presented in this paper utilizes a lightweight approach to promote an open and traceable system using blockchain technology. Storage, latency, and computational requirements can be reduced using our proposed model. The results from our simulation verify the security and efficiency of the proposed model, compared with traditional consensus approaches. As a future work, we aim to examine the trust model on real data of an IoT supply chain system.
